# Dietary Nutrients and Bioactive Substances Modulate Heat Shock Protein (HSP) Expression: A Review

**DOI:** 10.3390/nu10060683

**Published:** 2018-05-28

**Authors:** Carolina Soares Moura, Pablo Christiano Barboza Lollo, Priscila Neder Morato, Jaime Amaya-Farfan

**Affiliations:** 1 Protein Resources Laboratory, Food and Nutrition Department, Faculty of Food Engineering, University of Campinas (UNICAMP), Campinas 13083-862 São Paulo, Brazil; jaf@unicamp.br; 2 School of Health Sciences, Federal University of Grande Dourados, Dourados 79825-070, Mato Grosso do Sul, Brazil; pablo.christiano@gmail.com (P.C.B.L.); primorato@gmail.com (P.N.M.)

**Keywords:** bioactive peptide, obesity, whey protein

## Abstract

Interest in the heat shock proteins (HSPs), as a natural physiological toolkit of living organisms, has ranged from their chaperone function in nascent proteins to the remedial role following cell stress. As part of the defence system, HSPs guarantee cell tolerance against a variety of stressors, including exercise, oxidative stress, hyper and hypothermia, hyper and hypoxia and improper diets. For the past couple of decades, research on functional foods has revealed a number of substances likely to trigger cell protection through mechanisms that involve the induction of HSP expression. This review will summarize the occurrence of the most easily inducible HSPs and describe the effects of dietary proteins, peptides, amino acids, probiotics, high-fat diets and other food-derived substances reported to induce HSP response in animals and humans studies. Future research may clarify the mechanisms and explore the usefulness of this natural alternative of defense and the modulating mechanism of each substance.

## 1. Introduction

Living organisms are constantly exposed to various types of stress that tend to damage delicate cellular structures, thus avoiding eventual interference with vital cell functions. In response to such insults, the body naturally activates several mechanisms of protection, one of which is the heat-shock-protein (HSP) signaling system.

Evolutionarily, HSPs are a highly-conserved family of proteins that play a critical role in guiding both the initial folding of nascent proteins and the subsequent refolding of partially denatured structures, thus granting protection to cells against stressful environments. Previous studies have dealt mainly with the external or internal sorts of cell aggression that can induce HSP responses and the internal mechanisms that lead to cytoprotection. It has been proposed that HSP inducers might have their origin in food components such as proteins, amino acids, and peptides, among others. Thus, foods that have the potential to stimulate an HSP response could be viewed as vehicles of specific-health action operators or even as sources for the development of novel functional foods [1,2,3,4,5,6].

One of the most fascinating scientific aspects of feeding and metabolism that we can recognize is the multitude of functions that food components can perform besides what is classically understood as nourishment. Therefore, foods that have the potential to stimulate or induce the heat-shock response should deserve special attention because they may naturally trigger the production of defense molecules against current and probable environmental threats. This review will summarize the current state of knowledge on the relationship between diet, bioactive substances present in foods and the heat shock response.

## 2. Heat Shock Proteins

Ferruccio Ritossa first reported the cellular stress response when a curious increase occurred in chromosomal activity of *Drosophila* cells that had undergone heat treatments [7,8]. The phenomenon was termed “heat shock response” and 12 years later, the Ritossa experiment was related to heat shock protein (HSP) expression by Tissieres et al. (1974) [9]. HSPs were soon recognized as the physical entities directly responsible for this endogenous protein defense system that can be rapidly induced to repair damages occurring in other proteins, thereby preserving the cells’ integrity and granting stress tolerance against not only upward temperature variations, but a wide variety of aggressive agents.

Heat stress can lead to an initial step of the thermal denaturation of delicate proteins such as receptors, signal transducers, transporters and immune proteins. This should be followed by the loss of homeostasis in a cascade-fashion that worsens the ensuing adverse consequences. HSPs are involved in the proper folding of nascent proteins, rescuing proteins that were for some reason inadequately structured, and refolding those that lost their stress-sensitive spatial conformation. They are responsible for keeping polypeptides in the key conformation for their translocation through organelle and cell membranes, modulating protein-protein interactions and preventing the aggregation by intra and intermolecular contacts. HSPs can repair damaged proteins or help in the degradation of irreversibly damaged proteins [10,11,12,13,14,15,16].

In eukaryotic organisms, the expression of heat shock proteins is mediated by the heat shock factor (HSF), which requires activation prior to being translocated to the nucleus. HSF1 is the main regulator of HSP expression [17]. Each HSP is followed by a number that corresponds to its approximate molecular weight in kilodaltons such as HSP70, HSP90, HSP60, HSP25 and has a rather specialized function [14,18]. For future concerns about each HSP, see the reviews [10,11,14,19,20].

## 3. Effects of Amino Acids on HSPs

Some amino acids, both in vitro and in vivo, influence the expression of HSPs in several tissues, and in most cases, under different types of stress. Whether stress-mediated or not, free amino acids are food-derived compounds likely to activate the HSP system and bring about various beneficial effects to the cell. It is well established that glutamine induces increased expression of HSP70 [13,21,22,23,24], including when associated with exercise [25].

The known protective effect of glutamine supplementation may be directly related to the ability of this amino acid to increase the expression of HSPs. This hypothesis was sustained after silencing the HSP70 gene with the induction of sepsis and observing that glutamine supplementation did not reduce the activation of proinflammatory cytokines nuclear factor kappa (NF-kB), interleukin 6 and tumor necrosis factor (TNFα), i.e., injury markers in the lung. Therefore, the suggestion stands that glutamine’s protection is dependent on HSP70 expression promoting the defense and resistance properties of the cell [22].

Moreover, the HSP70-mediated glutamine protective potential is dose-dependent, as evaluated by the extent of the tissue damage (ischemia and reperfusion) in lung [21]. Stimulation of HSP70 expression could be an important means to obtain cytoprotection in the kidneys. Another study shows that the use of a single glutamine dose 30 min prior to the induction of severe diarrhoea could increase HSP25, 70 and 90 in the intestine, and reduce the incidence and severity of diarrhoea [26].

Other amino acids such as arginine, histidine, glutamate, proline, alanine, and glycine were individually tested for their ability to perform as glutamine, if tried at comparable concentrations. The data revealed that none of the amino acids tested separately could increase the HSP. However, it is noteworthy that this study was carried out in vitro, and therefore the cells had only the amino acid tested as the sole source of nutrient, not the dynamics of the body, since glutamate, for example, can be used for the synthesis of glutamine. Therefore, a second attempt was made using higher concentrations (10 mM) of glutamate. At this concentration, HSP25 was raised but to an extent lower than that obtained with glutamine at 4 mM [27].

The mechanism by which glutamine can induce HSP expression continues to be investigated, but by using in vitro experiments: Hamiel et al. [28] suggested that the ability of glutamine is associated with the biosynthesis pathway of hexosamine and its enzymes. This pathway is responsible for the glycosylation of transcription factors, as well as nuclear and cytoplasmic proteins.

If on the one hand it has been established that glutamine is able to induce HSPs in experimental animals and humans, including when associated with exercise [25], on the other hand, few studies report if other amino acids can induce the HSPs. An in vitro study by Baird et al. [29] showed that l-threonine induces HSP70 and HSP25 expression in intestinal epithelial heat-injured cells, thus diminishing apoptosis. Similarly, l-methionine supplementation has been reported to increase HSP70 in bovine mammary epithelial cells submitted to hyperthermia stress [30].

Supplementation with l-arginine, an indispensable amino acid for both new-born humans and young pigs, was also observed to increase HSP70 expression in the intestine of pigs [31]. Another study, however, reported that supplementation with arginine proved to preserve the integrity of muscle fibers, but brought no changes in either mRNA expression or HSP70 and HSP90 concentrations in exercised rats [32]. Under stress conditions produced by exercise, we have shown that oral supplementation with arginine increases muscle HSP70 and HSP90 expression in rats when compared to leucine, isoleucine or valine, but no effect was observed on HSP25. Additionally, arginine enhanced HSP levels to the same intensity as did glutamine [2]. 

Amino acid deprivation seems to limit the heat shock factor (HSF1) activity, which plays an important role in the synthesis of HSPs. Cells cultured with either leucine, lysine or glutamine deprivation show reduced HSF1, as well as HSPs mRNA levels. Thus, it is suggested that amino acid deprivation can compromise the defense capacity of the organism, since it may lead to the reduction of HSP synthesis [33].

## 4. Proteins and Bioactive Peptides

Until not long ago, classic nutrition would teach that food proteins were of vital importance for the growth and maintenance of an organism because of the quality and quantity of the amino acids they could deliver. Currently, it is established that proteins are also sources of peptides that can carry cell-signaling activities or specific health functions over and beyond the ultimate mission of delivering their amino acids for tissue building and repair. Food bioactive peptides have been defined in several ways, indicating that they are protein fragments with specific sequences in food proteins that can positively impact the state of health [34]. Here, we treat bioactive peptides as signaling protein molecules capable of triggering specific physiological responses in the body.

Therefore, mixtures of different proteins exhibiting a similar or nutritionally equivalent overall sum-total of amino acids (amino acid profile) may not necessarily grant the body equivalent health benefits. Nevertheless, the digestion of different types of dietary proteins has been shown to generate peptides with different effects on the body’s metabolism, including the expression of HSPs. We have shown, for instance, that consumption of peptides resulting from partially hydrolysing milk whey proteins increases the expression of HSPs. The specificity of some branched-chain amino acid-containing dipeptides indicates that Leu-Val, but not Val-Leu, has been found to increase the soleus muscle expression of HSP70, HSP25 and HSP90, whereas lle-Leu, but not Leu-Ile, elevates the muscle expression of HSP60, HSP70 and HSP70 in plasma. In addition, Leu-Val and lle-Leu peptides apparently affect HSPs in different and independent mechanisms, suggesting that Leu-Val signalizes functions in peripheral tissues affecting a greater number of muscle HSPs (HSP90, HSP70, HSP25), whereas lle-Leu appears to specifically influence both plasma and muscle HSP70, with potential systemic implications [1,3].

The consumption of whey protein in the hydrolyzed form (WPH), used as a source of protein in the diet, was able to increase the expression of HSP70 in lung, soleus and gastrocnemius muscles of rats subjected to thermal stress from exercise [5]. In addition, WPH also increased HSP90 expression and the co-chaperone activator of heat shock protein ATPase (Aha1), but without altering the expression of HSP25 or HSP60. Aha1 is known for accelerating conformational transitions and increasing the ATPase activity of HSP90 and is thus involved in folding processes mediated by HSP90 [4]. This ability, however, was not detectable when the dietary protein was fed either as intact whey protein or intact casein. Nevertheless, the most remarkable feature was that the whey hydrolysate was capable of inducing HSP70 expression even in the absence of stress [5].

In support of the proposed nutrient-mediated HSP induction, the work by Liedel et al. [35] showed that HSP70 can be induced in the small intestine of immature rat pups upon exposure to rat-mother’s milk. These authors proved that formula-fed premature pups were responsible for HSP70 mRNA reduction in ileum sections, compared to those fed the mother’s milk. We have elaborated further on the possible benefits of the bioactive milk whey protein peptides as HSP inducers, particularly in the case of type-2 diabetic patients that are impeded from doing physical exercise [36].

Supplementation with either l-glutamine or l-alanine, either in their free forms or l-alanyl-l-glutamine dipeptide, for 21 days equally upregulated tibialis anterior skeletal muscle HSP27 expression and reduced damage markers such as creatine kinase and TBARS in exercised rats [37].

**Figure 1:**
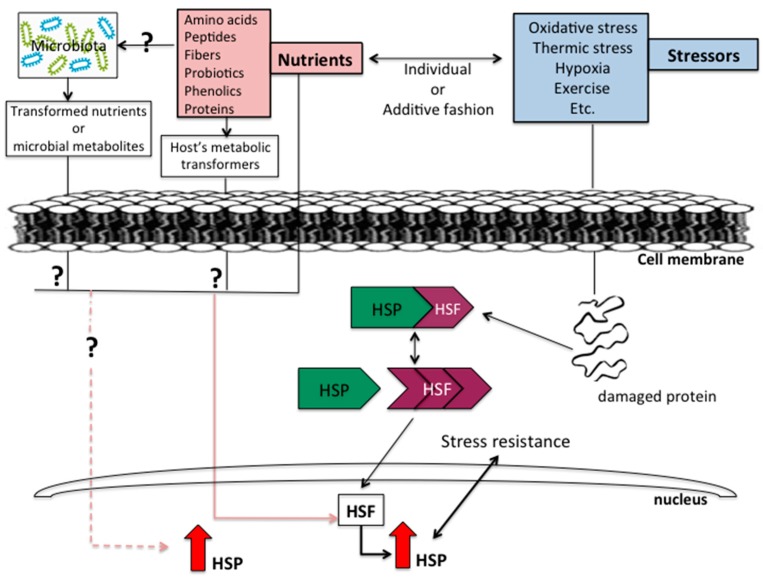
Schematic representation of the role of the classical heat shock-response inducers, now adding nutrients and bioactives. The two kinds of inducers could act individually or in an additive fashion. Explanation: (left side) an ingested nutrient or bioactive could enter directly both the cytoplasm and nucleus as such or be transformed by the microbiota (extracellularly to the host) or by the host’s (intracellularly) own metabolism. On the right-hand side, the classical scheme showing the induction of HSPs that requires the dissociation of the HSP-HSF complex and the participation of HSF in the production of HSP in the nucleus.

Figure 1 summarizes the interaction between the action of inducers and HSP response. Inducers can be either stressors or dietary nutrients, which could act individually or synergistically. A more integrated view of an HSP response that includes the plausible action of some nutrients could be as follows: (a) (on the basis of a nutrient or a bioactive) after consumption of specific nutrients, HSPs syntheses could be either mediated by HSF or directly act on the increase of HSPs, by mechanisms yet to be elucidated; (b) (on the basis of stressors) stress will trigger one or more of a yet partially defined mechanism. In addition, nutrients can still undergo metabolic modifications such as complexing with other compounds, glycosylation, hydrolysis or even involving the gut microbiota.

## 5. Dietary Fiber

Given that both the quality and quantity of dietary fiber can have marked effects on the intestinal microbiota and general health, it has been suggested that fibers may influence intestinal HSP expression. Intake of the the water-insoluble fraction of psyllium fiber for five days was found to increase intestinal HSP25 expression in mice, as determined by the increase in mRNA and protein levels [38]. This fiber, however, did not influence HSP70 in the jejunum, ileum or colon. The boost observed in HSP25 in the intestine correlated with a suppression of oxidant-induced malondialdehyde production. Although psyllium fiber induced HSP25, no difference was found in the HSF1 expression, suggesting that the upregulation of HSP25 after the consumption of the psyllium fiber occurs independently of its transcription factor—HSF1 [38]. These data could be contextualized with those of an earlier reported by Nishizawa et al. [39], reporting that HSF1-null mice were able to induce HSPs expression in soleus muscle. Although there is no definite explanation, it was suggested that the regulation of HSPs can be mediated by a yet unknown mechanism that does not involve HSF1. 

Similarly, Liu et al. [40] suggest that the intensity of HSP27 gut expression could be correlated to specific dietary compounds such as chicory fiber. These authors reported that consumption of a high-fiber diet based on chicory forage (80 g/kg) for 18 days enhanced HSP27 expression in the ileum and colon of pigs. The chicory root showed results resembling chicory forage but to a lower degree for HSP27 levels. 

## 6. Probiotics

The use of probiotics with the purpose of improving health parameters has grown worldwide. A probiotic is any microorganism capable of surviving the digestion process and that carries positive health benefits to the host. Some mechanisms that explain the benefits associated with the consumption of probiotics have been proposed. One such mechanism deals with the ability of probiotics to inhibit the nuclear factor-kB of the inflammatory pathway and at the same time induce the expression of HSP25 and HSP70 [41]. Petrof et al. [41] used a probiotic formulation containing *Streptococcus salivarius subsp thermophilus*, *Lactobacillus casei*, *L. plantarum*, *L. acidophilus*, *L. delbrueckii subsp. bulgaricus*, *Bifidobacteria longum*, *B. infantis*, and *B breve*, and observed an increase in colon HSP70 and HSP25. In contrast, we have reported that the consumption of a dessert with the addition of probiotic *L. acidophilus* to the formulation did not change the HSP70, HSP60 or HSP90 muscle expressions [42], thus suggesting that the quantity, type of probiotic (microorganisms), probiotic formulation and tissue analyzed can influence the induction capacity of HSP expression.

Tao et al. [43] reported the existence of active peptides (<10 kDa) in or derived from *Lactobacillus* GG that are responsible for stimulating the expression of HSP70 and HSP25 in mouse colon cells. The peptides are heat stable and have bioactivity at pH 4.0. The authors, however, did not identify any specific peptides. 

It is pertinent to note that there are variations in the composition and predominance of microorganisms making up a probiotic, and that this is a feature in the formulation of a probiotic food, which may affect the extent of the health effects. For one thing, involvement of the gut microbiota in the human metagenome is so great that the indirect action of many nutrients or bioactive substances on the heat shock response through the microbiota should not be ruled out [44].

## 7. Phenolic Compounds

Besides their widely studied antioxidant properties, many phenolic compounds display individual functions such as specific cell signaling competences, thus partly justifying the existence of such a large diversity of chemical structures encountered in nature. Resveratrol, a polyphenol typically found in wine, has been shown to affect the expression of HSP27, HSP70 and HSP90 in the spleen and thymus of chickens [45]. The authors found that this phenolic can either selectively stimulate or repress the expression of a chaperone, depending on the dose, the tissue and the presence or absence of heat stress. 

As far as the function of some sulfur compounds found in foods, Zhang et al. [46] have hypothesized that strategic free sulfhydryl groups (free sulfhydryls) in the sequence of HSF1 are modified by active sulfhydryl-containing compounds, such as sulforaphane and glutathione. According to these authors, this modification activates HSF1 thereby setting the cytoprotective HSP pathway into action. Since the same kind of sulfhydryl compounds also activate transcription controller KEAP1, they proposed that both the HSP70 and KEAP1/Nrf2/ARE pathways are set off simultaneously in a concerted fashion. Reports about other food sulfur compounds, such as allicins, follow the same standard approach focusing on the recovery power on tissue injury, rather than the protective effect granted by the bioactive substance. Consequently, in all cases the sham or injury-null control already contained the bioactive, so that the possibility of the substance alone eliciting the heat shock response was not detected.

Since HSPs tend to improve cell protection of even tumor cells, HSP inhibition should result in vulnerable tumor cells. Red wine, and particularly Syrah wine, that has a high phenolic content, has exhibited a high capacity to inhibit HSP70 and HSP27 in human tumor cells. In contrast, white wine, that has low phenolic contents, was reported not to influence HSP70 or HSP27 [47].

A protective effect of propolis as a modulator of the antioxidant system has been proposed mainly due to its high flavonoid content. The phenolic extract of propolis induced HSP70 expression in damaged testis of rats. The propolis extract has many different phenolic compounds mostly chrysin and vanillin, although it is not specifically known which compound or mixture is responsible for the increase in HSP [48]. Another phenolic compound with the capacity to induce HSP70 is curcumin, a natural pigment found in some plants. Exposure of frog A6 kidney cells to 10–50 μM of curcumin has been shown to enhance the HSP30 and HSP70 content and to improve thermotolerance [49]. 

Quercetin is a flavonoid known for its antioxidant and anticancer properties at the progression stage. Chander et al. [50] investigated the protective effect of quercetin against lead acetate neurotoxicity following oral administration of quercetin. The results showed that the toxic treatment upregulated HSP70 and neurological damage parameters while quercetin (100 mg/kg) was effective in reducing oxidative stress and neurological damage parameters and also decreased HSP70 expression in the cerebellum, cortex and hippocampus. 

## 8. High-Fat Diet and HSPs

Despite the fact that obesity and type 2 diabetes mellitus are among the most prevalent morbidities globally, the first observation linking HSPs and diabetes was based on data showing that insulin-resistant obese patients with type 2 diabetes had reduced muscle levels of HSP70 [51]. Subsequently, several studies also reported this reduction and/or deficiency in the muscle expression of HSPs in obese humans and animals [52,53,54,55]. It was suggested that the pathogenesis of type 2 diabetes could involve deficiency in the expression of genes that are primarily responsible for defence mechanisms, such as HSPs [54]. In diabetic monkeys, regulation of tissue-specific HSP was found, in which the monkeys had lower HSP70 in the blood and liver, besides reduced activation of HSF1. However, the pancreas had maintained levels of HSP, probably to protect the beta cells from the stress of insulin hypersecretion [56].

High-fat diet-induced obesity can reduce the expression of HSPs, while the increase and/or restoration of HSP expression can prevent or soften the damage caused by obesity induced by a hyperlipidic diet, promoting an improvement in the insulin sensitivity, glucose tolerance and inflammation reduction. An increase in muscle HSP70 could signify the prevention or amelioration of the damages caused by a high-fat diet-induced obesity, giving protection against disturbances in metabolic homeostasis, acting in the reduction of inflammation, improvement in oxidative capacity (cellular respiration) [51], reduction in insulin resistance [53], improvement in e glucose tolerance and increase in insulin signaling [57].

It is known that obesity stimulates the activation of inflammatory kinases such as c-Jun NH2-terminal kinase (JNK) and kB kinase-B (IKKβ) [51,53]. Activation of these kinases impairs insulin signaling pathways, in turn interfering with the phosphorylation of receptor substrate 1 (IRS1), reducing the interaction with downstream proteins such as PI3K and consequently reducing glucose uptake. The ability of HSP70 to inhibit phosphorylation and activation of JNK and IKKβ, which are abnormally high in obese animals, and upregulation of this chaperone has been suggested as a possible strategy to remediate glucose intolerance in obese individuals [6,57].

Thus, the ability of HSPs to inhibit the upregulated kinases in obesity is a suggested mechanism to decrease insulin resistance and improve glucose tolerance in hyperlipidic-diet-induced obese animals [6]. For this reason, external induction of HSP70 has been suggested to protect the body from insulin resistance and glucose tolerance in high-fat induced obesity [51]. The complexity of a hyperlipidic diet, however, may make this approach not as simple as it looks. Recently we have shown, for instance, that mice consuming a hyperlipidic diet with a high content of oil (mixture of lipid factions) in the absence of lard reduced muscle HSP25 expression if consumed with casein as a source protein, while WPH preserved HSP25 levels. However, the hyperlipidic diet with a high content of trans-fats from partially hydrogenated oils did not cause any changes in HSP25, HSP60, HSP90 and HSP70 expression with casein or WPH [58]. 

Although the protective effect of intramuscular HSP70 and HSP25 against insulin resistance, glucose intolerance and hyperinsulinemia in obese individuals induced by a high-fat diet has been established, little is known about the effects of extracellular HSPs and their behavior on obesity [6,51,52,55].

It was postulated that HSPs were exclusively intracellular components, but these proteins circulate outside the cells. The release of HSPs into circulation was associated with cell disruption, justifying their presence in the extracellular environment. However, experiments with controlled or inhibited lysis showed that the release of HSPs into circulation occurred even in the absence or independent of cell death, suggesting that the release of HSPs occurs by active mechanisms; the exosomes are one of the more accepted mechanisms of secretion [9]. Although the elucidation of the HSP extracellular functions in health and disease has not been completely established, it has been recognized that intracellular HSPs exert protective anti-inflammatory effects through extracellular active pro-inflammatory pathways [52].

Gram-negative bacterial components are known to bind to and are recognized by the Toll like receptor (TLR4), but other circulating molecules of endogenous origin may be recognized by TLR4 [59,60]. The extracellular HSP60 binds to TLR4, signaling the production and secretion of pro-inflammatory components such as interleukins and TNFα, mediating inflammation and insulin sensitivity [60,61]. According to Marker et al. (2012) [62], plasma HSP60 is higher in obese than in lean individuals, and adipocytes release HSP60 by increasing their circulating levels by activating the inflammatory pathway through the TLR4 receptor, promoting insulin resistance. Similarly, obese subjects with type 2 diabetes also have elevated circulating levels of HSP60 [63]. It has recently been reported that the weight-loss process after bariatric surgery reduces extracellular levels of HSP60, known to be elevated in morbidly obese individuals [64]. 

For extracellular HSP70 levels, the literature has divergences. Islam and authors (2014) [65] showed that the concentration of extracellular HSP70 is inversely correlated with obesity, body fat percentage, waist circumference, and insulin resistance. In contrast, obese individuals [63] and those with type 2 diabetes have high levels of circulating HSP70 and may serve as a new marker for disease progression or duration [66].

Table 1 summarizes the dietary components and their effects on HSPs.

## 9. Other Foods and Nutrients

### 9.1. Chia

Chia (*Salvia hispanica* L.) is a South American oilseed considered also as a source of protein, polyphenolic compounds and dietary fiber. The ingestion of chia oil replacing soybean oil for 6 and 12 weeks increased muscle HSP70 and HSP25 expression in high-fat-high-fructose diet-induced obese mice. Under similar conditions, chia seed consumed for 6 weeks also enhanced HSP70, but not HSP25 expression [67]. 

### 9.2. Copper

Dietary copper deficiency has been reported to reduce myocardial HSP70 and HSP60 mRNA but did not influence HSP90. The outstanding reduction of HSP60 in the atria reflects the observed alterations in mitochondrial function and structure [68].

### 9.3. Garlic

Allicin is a sulfur component present in garlic with relevant positive effects on health. Treatment of spinal cord-injured mice with allicin via intraperitoneal injection has been shown to enhance HSP70 protein levels in the damaged region, as well as other positive effects. The best allicin dose to induce HSP was 10 mg/kg for mice [69].

### 9.4. Dietary Restriction

Dietary or caloric restriction is understood as the limitation of the intake of a complete or balanced diet. Such a procedure is often mentioned as a strategy to promote longevity in mammals [72]. 

Key nutrient restrictions that block biochemical routes limiting the rate or halting the synthesis of inducing factors can be considered as a kind of dietary stress that lowers HSP expression. However, caloric restriction has been reported to revert the age-dependent decreased capacity of rodents to express HSP70, as determined by the longer survival of hepatocytes from old mice [73]. Moore et al. [70] reported that dietary restriction favors HSP70 induction in rat alveolar macrophages even in old mice, thus suggesting that dietary restriction may improve HSP response and favor the cytoprotective effect. Similar work showed improved HSP70 expression with a 30–40% caloric restriction in *Caenorhabditis elegans* [71].

## 10. Conclusions

That the presence of some nutrients and bioactive substances favors or enhances the display of a heat shock response is something readily accepted if the organism is under stress, but that a nutrient or a bioactive by itself being capable of inducing the expression of HSPs without the intervention of stress is an emerging notion that may deserve deeper investigation. The experimental studies reviewed here highlight the direct connection that exists between some nutrients and the modulation of HSPs, between some bioactive substances and the possible metabolic targets where they can exert their protective functions, but research aiming to show any independence between HSP induction and a state of preexisting stress is scarce. Specific foods can be used as new strategies to modulate HSP expression with the goal of improving health. Therefore, it seems reasonable to think that by selecting the proper kinds of dietary foods the body may be tapping external sources of bioactive substances which will act as cell signalers for the induction of HSPs, probably at maintenance levels, when the organism is under an apparent metabolic steady-state. Future studies could search for other food bioactives with this capacity and aim at elucidating the specific mechanisms of inducing the expression of HSPs.

## Figures and Tables

**Table 1:** Studies involving dietary components in the regulation of HSPs.

Dietary Nutrients	Effect on HSP	Tissue/Cell	References
Glutamine	Increase HSP70, HSP25, HSP90	Lung, intestine, intestinal cell, blood mononuclear cells	[21,22,24,25,26]
l-threonine	Increase HSP70, HSP25	Intestinal epithelial cells	[29]
l-methionine	Increase HSP70	Mammary epithelial cells	[30]
l-arginine	Increase HSP70	Intestine	[31]
l-arginine	None change HSP70, HSP90	Muscle	[32]
l-arginine	Increase HSP70, HSP90; none change HSP25	Muscle	[2]
Leu-Val dipeptide	Increase HSP70, HSP90, HSP25	Muscle	[1,3]
lle-Leu dipeptide	Increase HSP70, HSP60, extracellular HSP70	Muscle/plasma	[1,3]
Whey protein hydrolysate	Increase HSP70, HSP90; none change HSP60, HSP25	Lung and muscle	[4,5]
Formula for premature pups	Reduce HSP70	Intestine	[35]
l-glutamine, l-alanine or alanyl-glutamine dipeptide	Increase HSP27	Muscle	[37]
Psyllium fiber	Increase HSP25; none change HSP70	Intestine	[38]
Chicory fiber	Increase HSP27	Intestine	[40]
Probiotic formulation	Increase HSP70, HSP25	Colon	[41]
Probiotic (*L. acidophilus*)	None change HSP60, HSP90, HSP70	Muscle	[42]
Probiotic (Lactobacillus GG)	Increase HSP70, HSP25	Colon cell	[43]
Phenolic (Syrah red wine)	Inhibit HSP70, HSP27	Tumor cells	[47]
White wine	None change HSP70, HSP27	Tumor cells	[47]
Própolis	Increase HSP70	Testis	[48]
Curcumin	Increase HSP30, HSP70	A6 kidney cells	[49]
Quercetin	Reduce HSP70	Cerebellum, cortex and hippocampus	[50]
High-fat diet (lard)	Reduce HSP70	Muscle	[52,53,54,55]
Hyperlipidic oil diet	Reduce HSP25	Muscle	[58]
Hyperlipidic high trans-fats content diet	None change HSP25, HSP60, HSP90, HSP70	Muscle	[58]
High-fat diet	Increase extracellular HSP60	Plasma	[62]
Chia oil with high-fat-high-fructose	Increase HSP70, HSP25	Muscle	[67]
Copper deficiency	Reduce HSP70, HSP60; none change HSP90	Myocardial	[68]
Allicin	Increase HSP70	Spinal cord	[69]
Dietary restriction	Increase HSP70	Alveolar macrophages	[70]
Caloric restriction	Increase HSP70	Caenorhabditis elegans	[71]
